# Retraction: SSRP1 Promotes Colorectal Cancer Progression and is Negatively Regulated by miR‐28‐5p

**DOI:** 10.1111/jcmm.70048

**Published:** 2024-09-12

## Abstract

**Retraction**: Wu W, He K, Guo Q, et al. SSRP1 Promotes Colorectal Cancer Progression and is Negatively Regulated by miR‐28‐5p. *J Cell Mol Med*. 2019;23(5): 3118‐3129. doi: 10.1111/jcmm.14134

The above article, published online on 14 February 2019 in Wiley Online Library (wileyonlinelibrary.com), has been retracted by agreement between the authors; the Journal Editor‐in‐Chief, Stefan Constantinescu; The Foundation for Cellular and Molecular Medicine; and John Wiley & Sons Ltd. The retraction has been agreed due to the authors' admission that some of the images were mistakenly included in this article, with some of the data belonging to another project. Additionally, the article was submitted for publication by Wei Wu without consent from the co‐author Honggang Yu.

**Retraction**: Wu W, He K, Guo Q, et al. SSRP1 Promotes Colorectal Cancer Progression and is Negatively Regulated by miR‐28‐5p. *J Cell Mol Med*. 2019;23(5): 3118‐3129. doi: 10.1111/jcmm.14134

The above article, published online on 14 February 2019 in Wiley Online Library (wileyonlinelibrary.com), has been retracted by agreement between the authors; the Journal Editor‐in‐Chief, Stefan Constantinescu; The Foundation for Cellular and Molecular Medicine; and John Wiley & Sons Ltd. The retraction has been agreed due to the authors' admission that some of the images were mistakenly included in this article, with some of the data belonging to another project. Additionally, the article was submitted for publication by Wei Wu without consent from the co‐author Honggang Yu.

